# Association of Ischemia Localization with Recorded PCI Procedural Category in Patients Undergoing Myocardial Perfusion Imaging

**DOI:** 10.3390/diagnostics16182978

**Published:** 2026-09-15

**Authors:** Ozan Kandemir, Hakkı Kaya, Emrah Doğan

**Affiliations:** 1 Department of Nuclear Medicine, Faculty of Medicine, Sıtkı Kocman University, 48000 Mugla, Turkey; 2 Department of Cardiology, Eighteen March University, 17100 Çanakkale, Turkey; 3 Department of Radiology, Polyclinique Temara, Temara 12000, Morocco

**Keywords:** myocardial perfusion imaging, coronary artery disease, myocardial ischemia, percutaneous coronary intervention, procedural category, stent

## Abstract

**Background**: Myocardial perfusion imaging (MPI) plays a central role in the noninvasive evaluation of myocardial ischemia and in guiding clinical management in patients with suspected or known coronary artery disease. While ischemic burden is an established component of MPI interpretation, the relationship between ischemia localization and the procedural strategy recorded among patients undergoing percutaneous coronary intervention (PCI) remains less well defined. This study aimed to evaluate whether the anatomical localization of ischemia identified by MPI was associated with the two PCI procedural categories recorded in the study dataset (Stent versus PTCA + stent). **Methods**: In this retrospective cohort study, 328 patient records were assessed for eligibility and 268 patients with complete MPI localization and treatment data were included. Seven patients received medical therapy alone and 261 underwent percutaneous coronary intervention (PCI); no patient underwent coronary artery bypass grafting (CABG). Within the PCI subgroup, the mutually exclusive patient-level source labels were Stent (including capitalization variants) and PTCA + STENT; no PTCA-only category was present. Because of the small medical-therapy group, inferential analyses were restricted to the 261 PCI patients. The overall association between the 13 ischemia–localization categories and PCI procedural category was assessed using the Fisher–Freeman–Halton exact test with Monte Carlo estimation. Multivariable logistic regression was performed with Stent as the dependent category and age, sex, anterior involvement, and multiregional ischemia as explanatory variables. **Results**: Among the 261 patients undergoing PCI, 144 were recorded in the Stent category and 117 in the PTCA + stent category. Across all 13 ischemia–localization categories, no statistically significant overall association was observed between ischemia localization and PCI procedural category (Fisher–Freeman–Halton exact test with Monte Carlo estimation, *p* = 0.7843). In multivariable analysis, anterior involvement (adjusted OR 1.02, 95% CI 0.58–1.82; *p* = 0.935) and multiregional ischemia (adjusted OR 0.69, 95% CI 0.33–1.41; *p* = 0.304) were not significantly associated with procedural category. Age was also not significantly associated with procedural category (adjusted OR 1.00, 95% CI 0.97–1.03; *p* = 0.993). Male sex was associated with lower adjusted odds of being in the Stent category relative to PTCA + stent (adjusted OR 0.40, 95% CI 0.23–0.70; *p* = 0.0012). **Conclusions**: In this cohort of patients undergoing PCI, ischemia localization was not significantly associated with the recorded procedural category (Stent versus PTCA + stent). The observed association with sex was secondary and should be interpreted cautiously. Given the very small medical-therapy group, no reliable conclusions can be drawn from this cohort regarding the likelihood of PCI versus medical management or comparative treatment efficacy.

## 1. Introduction

Coronary artery disease (CAD) remains a major cause of morbidity and mortality, and contemporary guidelines emphasize structured risk assessment and evidence-based management in patients with suspected or established CAD [1,2,3]. In this setting, myocardial perfusion imaging (MPI) has an important role in the non-invasive assessment of myocardial ischemia and in guiding clinical management decisions [4,5,6].

Current guidelines emphasize integrating functional ischemia assessment with anatomical and clinical information, particularly in patients with chronic coronary syndromes [1]. MPI provides valuable diagnostic and prognostic information by evaluating the presence, extent, and severity of inducible ischemia [4,6]. Observational data have also demonstrated that ischemic burden is associated with prognosis and may modify the relationship between medical therapy and revascularization [7,8].

However, the anatomical localization of ischemia may also be relevant to clinical interpretation and procedural planning. The standardized 17-segment model provides a common framework for regional localization, but the correspondence between myocardial segments and individual coronary arteries is variable rather than absolute [9,10]. Whether regional ischemia patterns are associated with procedural choices among patients already selected for PCI remains less well defined.

The ISCHEMIA trial further influenced the management of stable ischemic heart disease by demonstrating that an initial routine invasive strategy did not uniformly reduce major ischemic clinical events compared with an initial conservative strategy in patients with moderate or severe ischemia [11]. Taken together, randomized evidence and observational MPI studies reinforce the importance of interpreting ischemia in clinical context rather than using ischemia alone as a procedural mandate [7,8,11]. Regional perfusion information may nevertheless contribute to individualized interpretation, particularly when integrated with coronary anatomy [10,12,13].

Although several studies have investigated the prognostic significance of MPI abnormalities, relatively few have directly evaluated how regional perfusion information relates to subsequent procedural decision-making [12]. In the present retrospective dataset, PCI procedures were recorded in two patient-level categories, Stent and PTCA + stent; the clinical meaning of these broad source-documentation categories therefore requires cautious interpretation.

Therefore, the aim of the present study was to investigate the association between ischemia localization detected by MPI and the recorded percutaneous procedural strategy (Stent versus PTCA + stent) among patients undergoing PCI.

## 2. Materials and Methods

### 2.1. Study Design and Population

This retrospective cohort study evaluated patients who underwent myocardial perfusion imaging (MPI) at the Nuclear Medicine Clinic of Sivas Numune Hospital between January 2013 and January 2015 for suspected or known coronary artery disease.

The source screening file available for this retrospective study contained 328 patient records. Patients with missing or non-evaluable ischemia localization (*n* = 41), incomplete essential patient or demographic data (*n* = 16), or missing treatment information (*n* = 3) were excluded. After these exclusions, 268 patients with complete MPI localization and treatment data constituted the final study population. The available source file did not permit reconstruction of a larger pre-screening population beyond these 328 records.

**Figure 1:**
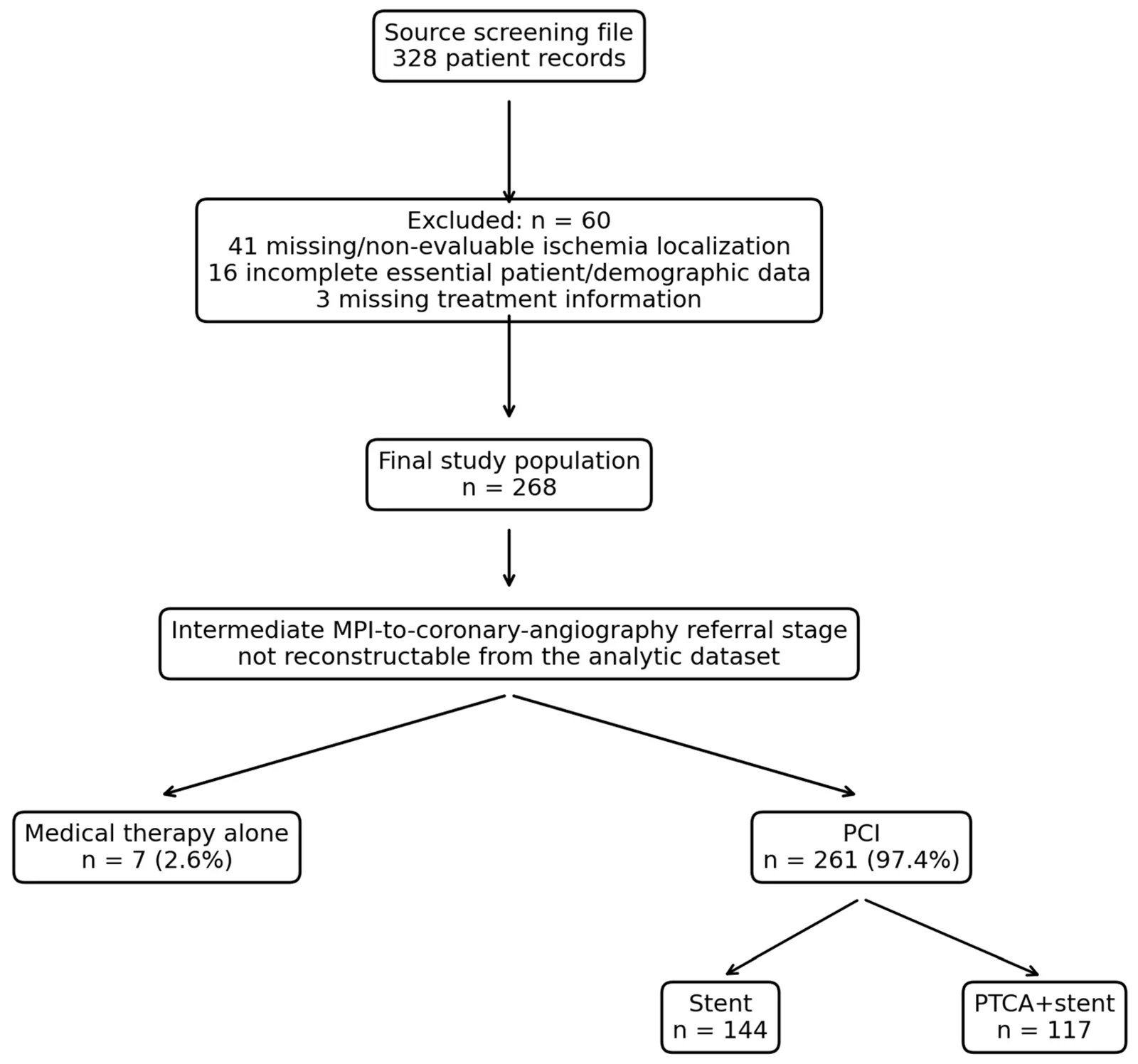
Patient selection and study flow. The intermediate pathway from MPI to coronary angiography could not be reconstructed because referral/angiography variables were not systematically represented in the analytic dataset. The final inferential analysis was restricted to the 261 patients undergoing PCI.

Demographic characteristics, including age and sex, ischemia localization, and subsequent treatment information were obtained retrospectively from hospital records. Coronary angiography was not an explicit eligibility variable in the reconstructed analytic dataset. A distinct normal/no-ischemia MPI category and the intermediate referral pathway from MPI to coronary angiography were not systematically represented in the analytic file; therefore, these stages could not be reconstructed reliably. The patient-selection process is summarized in Table 1 and Figure 1.

A total of 328 patient records were available in the source screening file. Sixty records were excluded because of missing or non-evaluable ischemia localization (*n* = 41), incomplete essential patient or demographic records (*n* = 16), or missing treatment information (*n* = 3). The final study population therefore consisted of 268 patients with complete MPI localization and treatment data. Of these, 7 patients (2.6%) were managed with medical therapy alone and 261 patients (97.4%) underwent PCI. The source analytic file did not allow reliable reconstruction of a separate normal-MPI category or of the intermediate MPI-to-coronary-angiography referral stage.

### 2.2. Assessment Protocol

The institutional protocol used a one-day stress–rest 99mTc-sestamibi MPI sequence. Patients fasted for at least four hours. When clinically appropriate and in the absence of contraindications, beta-blockers and calcium-channel blockers were withheld for 48 h before diagnostic testing. Exercise stress was performed using the modified Bruce protocol, with a target of at least 85% of the age-predicted maximum heart rate [(220 − age) × 0.85]. Stress testing was terminated according to clinical or electrocardiographic criteria, including limiting symptoms, significant arrhythmia or conduction disturbance, marked ST-segment change, hypotensive response, or severe hypertension. At adequate stress, 296–370 MBq of 99mTc-sestamibi was administered intravenously and exercise was continued for approximately one minute. Patients unable to exercise underwent dipyridamole stress (0.14 mg/kg/min for 4 min), followed by tracer administration. Stress imaging was performed approximately 30 min after exercise or dipyridamole stress. Approximately three hours later, 814–925 MBq of 99mTc-sestamibi was administered for the resting phase, and rest imaging was performed 45–60 min later. Images were acquired in the supine position using a Siemens Symbia S gamma camera (Erlangen, Germany) with an LEHR collimator, a 64 × 64 matrix, 64 frames, 25 s/frame for stress images, and 20 s/frame for rest images.

Additional technical metadata required for a fully reproducible SPECT protocol—including projection arc, energy-window specification, reconstruction algorithm and filter parameters, and ECG-gating status—were not systematically retained in the analytic dataset. Structured reader-level variables, including the number of interpreting physicians, blinding to subsequent treatment, and inter-reader agreement, were likewise unavailable and were not reconstructed retrospectively. For patients undergoing dipyridamole stress, a structured variable documenting pre-test caffeine or methylxanthine abstinence was also not retained in the analytic dataset; this aspect of patient preparation therefore could not be reconstructed retrospectively.

**Figure 2:**
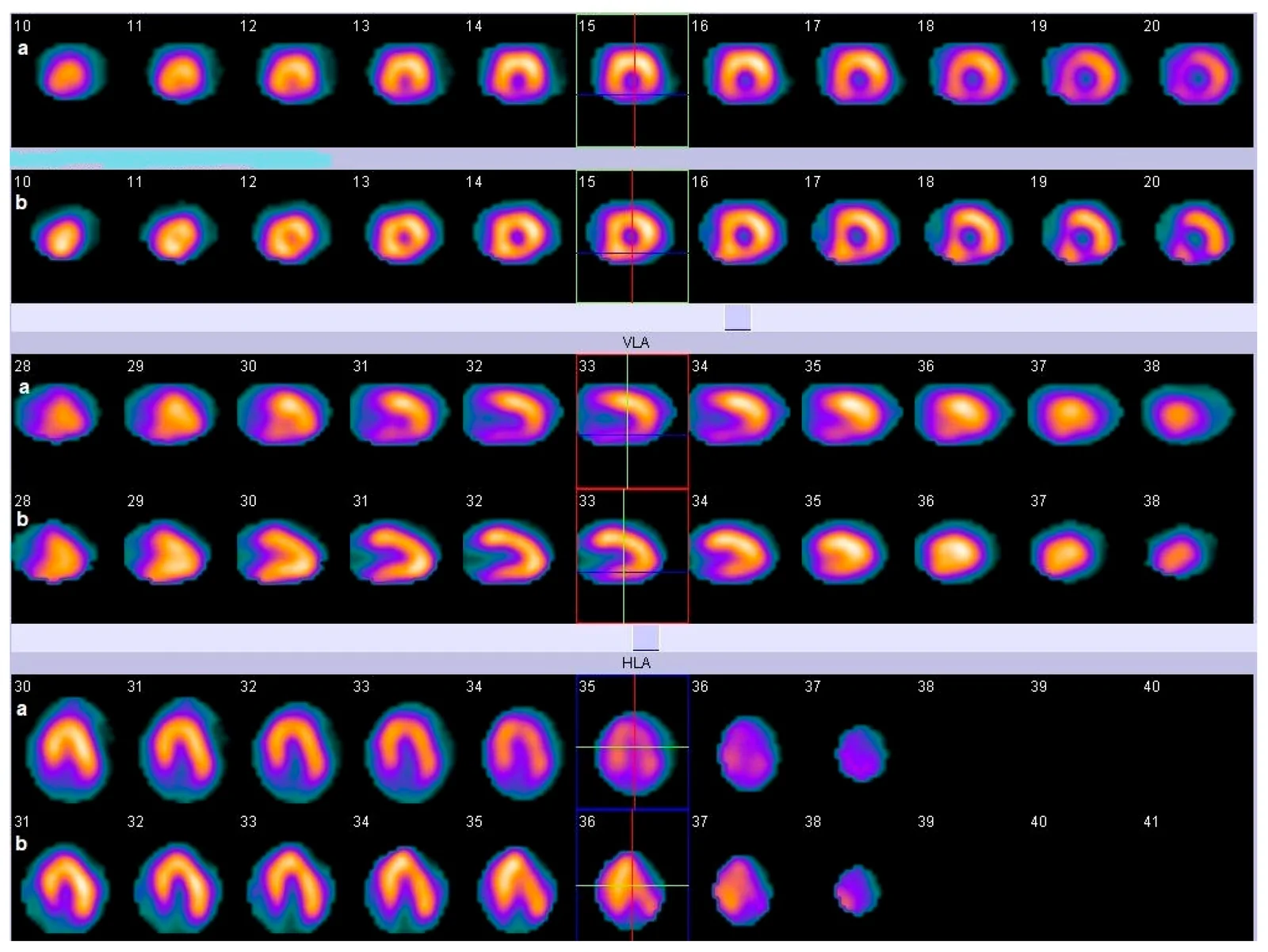
Illustrative case of stress-rest MPI (myocardial perfusion imaging). In a patient undergoing myocardial perfusion imaging, reduced activity was observed in the inferior wall across three different slices during stress imaging (**a**), whereas increased activity was observed in the same region across three different slices during rest imaging (**b**); the initial clinical assessment indicated inferior wall ischemia.

A 17-segment myocardial map was used for visual localization of perfusion abnormalities. Standardized segment-level stress and rest scores (e.g., 0–4 scoring) and the derived summed stress score (SSS), summed rest score (SRS), and summed difference score (SDS) values were not available in the retrospective analytic dataset. Accordingly, ischemic burden was not quantitatively assessed, and the present analysis was restricted to ischemia localization rather than quantitative ischemia severity (Figure 2).

### 2.3. Myocardial Perfusion Imaging and Ischemia Localization

Ischemic regions detected on MPI were classified according to the territorial categories recorded in the source dataset. Thirteen patient-level source categories were present in the final analytic dataset: anterior, anteroseptal, inferior, inferolateral, anterior + inferior, apex, apex + inferior, lateral, anterolateral, anterior + apex, inferoseptal, ALL, and anterolateral + apex. These categories were derived from the original clinical documentation and were not created by a new post hoc aggregation of stored 17-segment scores; individual segment-level data were not available for remapping. For regression analysis, anterior involvement was defined as any source category explicitly containing an anterior, anteroseptal, or anterolateral component. Multiregional ischemia was defined as combined territorial categories (categories containing “+”) or the source label ALL. The label ALL was retained as an undecomposed original multiregional category. Because its exact segmental composition could not be reconstructed from the analytic file, it was not recoded post hoc as anterior involvement.

Territorial classifications were derived from the original clinical MPI documentation and were accepted as recorded in the source dataset. The retrospective dataset did not contain dedicated variables indicating whether a suspected anterior/breast or inferior/diaphragmatic attenuation defect had been resolved by attenuation correction, prone imaging, gated wall-motion assessment, or another standardized artifact-adjudication method. Accordingly, attenuation artifacts could not be retrospectively re-adjudicated, and no post hoc reclassification of the original territorial interpretations was attempted. This limitation was considered when interpreting regional associations.

### 2.4. Treatment Strategy

Treatment strategies recorded after MPI were categorized as:

- Medical therapy alone
- Percutaneous coronary intervention (PCI)

No patient in the final cohort underwent CABG or other surgical revascularization; all invasive procedures represented in this cohort were PCI. Within the PCI subgroup, the source spreadsheet contained mutually exclusive patient-level procedural labels “Stent”/”STENT” and “PTCA + STENT”. Capitalization variants of Stent were harmonized into one Stent category, whereas PTCA + STENT was retained as a distinct source category. The final analytic dataset contained no PTCA-only category; therefore, PTCA + stent must not be interpreted as balloon-only angioplasty. The source data did not provide lesion-level definitions sufficient to determine whether the Stent label represented direct stenting or whether PTCA + stent represented a standardized procedural sequence. Accordingly, both groups are treated strictly as source-recorded procedural categories. Each PCI record was assigned to the single patient-level procedural label recorded in the source dataset, and records labeled PTCA + STENT were not reclassified into either a PTCA-only or a Stent-only group. Vessel-level procedural sequences, the number of treated vessels, and mixed multivessel treatment details were not systematically available; consequently, patients with multivessel intervention, when present, remained classified according to the single patient-level source label. Because only 7 of 268 patients received medical therapy alone, this group was retained for descriptive reporting but excluded from inferential comparisons of procedural strategy. The primary comparative analysis therefore focused on the 261 patients undergoing PCI and evaluated Stent versus PTCA + stent as recorded in the source data.

### 2.5. Statistical Analysis

Continuous variables were summarized using appropriate descriptive statistics. Because the medical-therapy group was very small (*n* = 7), inferential analyses of procedural strategy were restricted to the PCI subgroup. Age distributions between PCI procedural groups were compared using the Mann–Whitney U test, and sex distributions were compared using Fisher’s exact test. The overall association between ischemia localization and PCI procedural category was evaluated on the complete 13 × 2 contingency table. Because 50% of expected cell counts were below 5 (minimum expected count 0.90), the Fisher–Freeman–Halton exact test with Monte Carlo estimation was used as the primary test rather than the conventional Pearson chi-square test. Monte Carlo estimation used 500,000 fixed-margin random tables, random seed 20260816, and the two-sided Fisher criterion based on tables with conditional probability less than or equal to that of the observed table. Cramer’s V was reported as a descriptive effect-size measure for the 13 × 2 table. Binary logistic regression was performed with Stent as the dependent category (Stent = 1, PTCA + stent = 0). Because the revised analysis was exploratory, covariates were selected on clinical relevance and availability rather than by data-driven significance testing: age and sex were included as demographic covariates, and anterior involvement and multiregional ischemia as imaging covariates. A post hoc sensitivity analysis recoded the source label ALL as anterior involvement while retaining its multiregional classification. Results were reported as adjusted odds ratios (ORs) with 95% confidence intervals (CIs). No region-specific post-hoc significance tests were performed after the global exact test. All tests were two-sided and *p* < 0.05 was considered statistically significant. Analyses were performed in Python 3.13.5 using SciPy 1.17.0 and Statsmodels 0.14.6.

## 3. Results

### 3.1. Patient Characteristics

A total of 268 patients constituted the final study population. The overall median age was 67.0 years [IQR 60.0–73.0], and 150 patients (56.0%) were male (Table 2). Seven patients received medical therapy alone; their median age was 68.0 years [IQR 65.5–71.5], and 5 (71.4%) were male. The remaining 261 patients underwent PCI, including 144 patients recorded in the Stent category and 117 in the PTCA + stent category. Within the PCI subgroup, age distributions were similar between the Stent and PTCA + stent groups (median 67.5 years [IQR 60.0–73.3] versus 66.0 years [IQR 60.0–73.0], respectively; Mann–Whitney U = 8652, *p* = 0.707). Male patients accounted for 45.8% (66/144) of the Stent group and 67.5% (79/117) of the PTCA + stent group (Fisher’s exact *p* = 0.00047). Variables such as diabetes mellitus, hypertension, dyslipidemia, smoking status, prior myocardial infarction, left ventricular ejection fraction, and other cardiovascular risk factors were not systematically available in the analytic dataset and therefore could not be reliably summarized.

### 3.2. Ischemia Localization and Treatment Strategy

The primary inferential analysis was restricted to the 261 patients who underwent PCI. Ischemia localization was evaluated in relation to the recorded procedural category (Stent versus PTCA + stent); the 7 patients receiving medical therapy were excluded from this comparison because of the small sample size.

Of the 268 patients included in the study, 144 (53.7%) were recorded in the Stent category, 117 (43.7%) in the PTCA + stent category, and 7 (2.6%) received medical therapy alone. Table 3 presents all 13 ischemia-localization categories and accounts for the complete cohort (*n* = 268).

**Figure 3:**
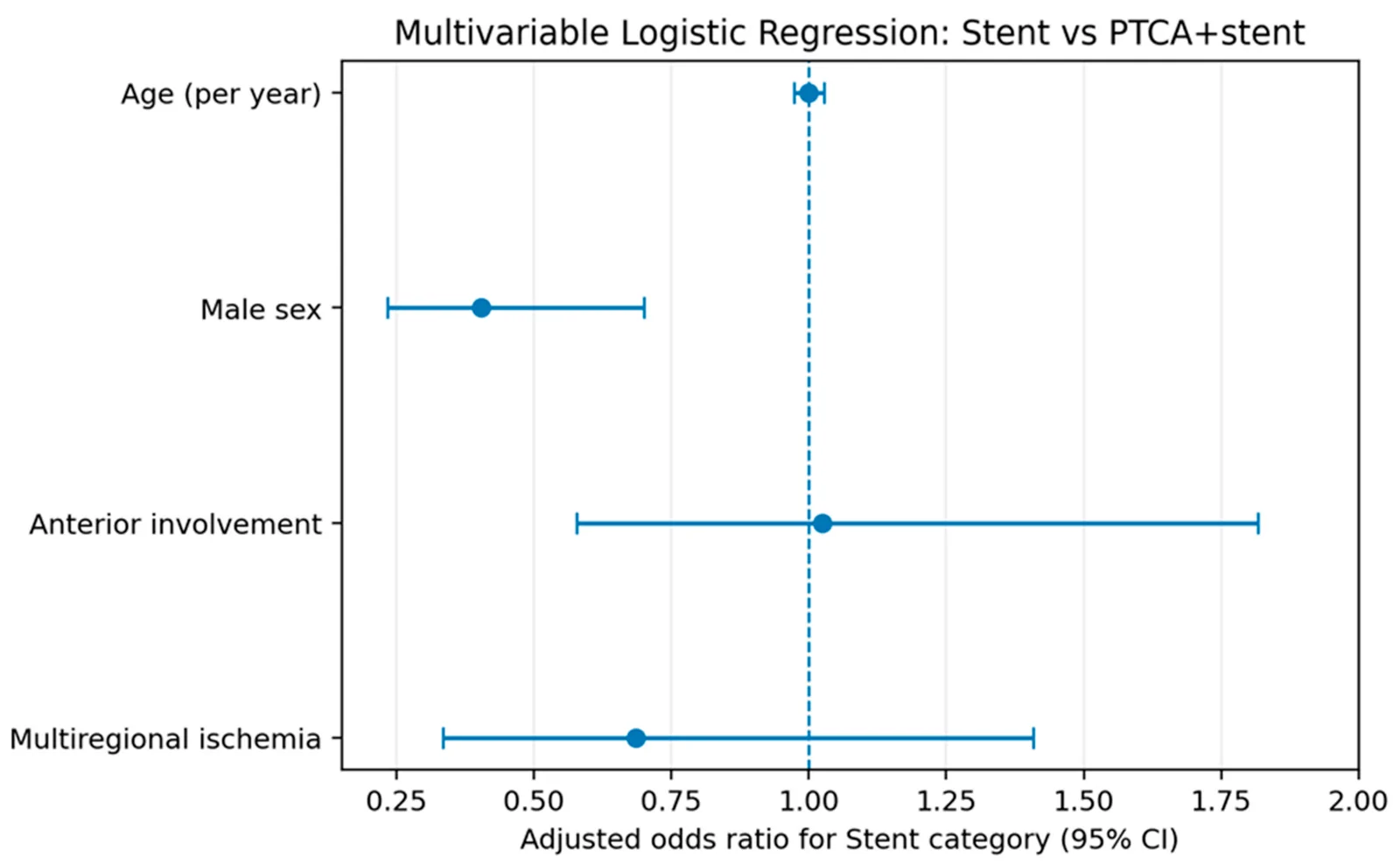
Multivariable logistic regression for the recorded PCI procedural category (Stent = 1, PTCA + stent = 0). Male sex was associated with lower adjusted odds of the Stent category (adjusted OR 0.40, 95% CI 0.23–0.70; *p* = 0.0012). Age (adjusted OR 1.00, 95% CI 0.97–1.03; *p* = 0.993), anterior involvement (adjusted OR 1.02, 95% CI 0.58–1.82; *p* = 0.935), and multiregional ischemia (adjusted OR 0.69, 95% CI 0.33–1.41; *p* = 0.304) were not statistically significant.

The complete 13 × 2 PCI contingency table yielded a Pearson chi-square statistic of 8.29 (12 degrees of freedom; *p* = 0.762). However, because half of the expected cell counts were below 5, the Pearson test assumptions were not satisfied. The primary inference was therefore based on the Fisher–Freeman–Halton exact test with Monte Carlo estimation, which showed no statistically significant association between ischemia localization and recorded PCI procedural category (*p* = 0.7843). The corresponding descriptive Cramer’s V was 0.18. In the post hoc sensitivity analysis that treated the source label ALL as anterior involvement, the conclusions were unchanged: anterior involvement remained non-significant (adjusted OR 1.03, 95% CI 0.57–1.86; *p* = 0.924), as did multiregional ischemia (adjusted OR 0.68, 95% CI 0.33–1.43; *p* = 0.313) (Figure 3).

**Figure 4:**
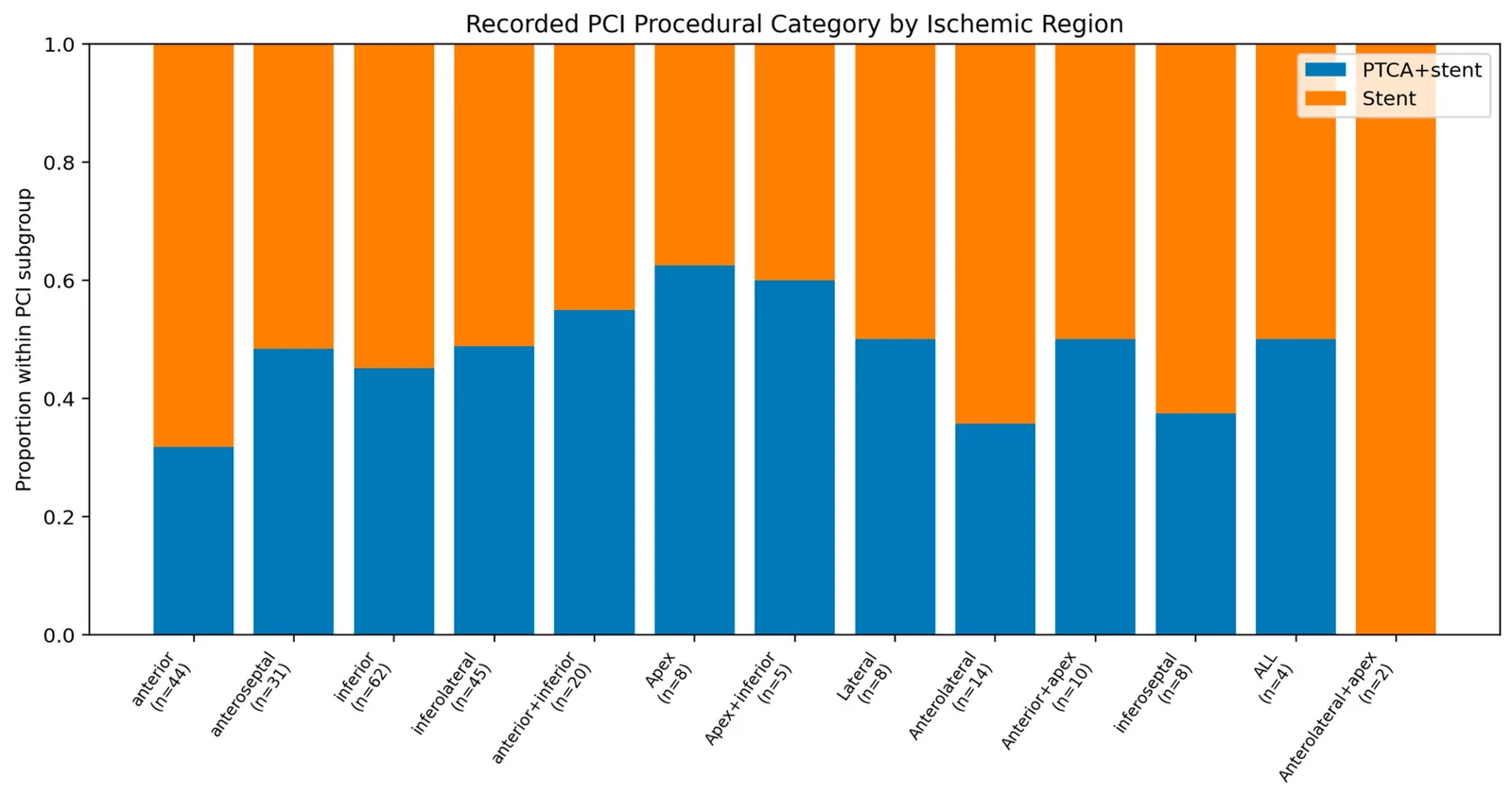
Recorded PCI procedural category by ischemic region. Proportional distribution of PTCA + stent and Stent categories among patients undergoing PCI; medical-therapy patients are not included in the plotted proportions. Category labels include the number of PCI patients contributing to each bar.

The overall association between the 13 ischemia-localization categories and the recorded PCI procedural category was not statistically significant on the Fisher–Freeman–Halton exact test with Monte Carlo estimation (*p* = 0.7843) (Figure 4). The conventional Pearson chi-square result was chi-square (12) = 8.29, *p* = 0.762, but was not used as the primary inference because 13 of 26 expected cells (50%) were below 5. The descriptive Cramer’s V was 0.18.

Multivariable logistic regression adjusted for age, sex, anterior involvement, and multiregional ischemia. Male sex was associated with lower odds of the Stent category relative to PTCA + stent (adjusted OR 0.40, 95% CI 0.23–0.70; *p* = 0.0012), whereas age, anterior involvement, and multiregional ischemia were not statistically significant.

## 4. Discussion

In this retrospective cohort study of 268 patients undergoing myocardial perfusion imaging (MPI), 7 patients received medical therapy alone and 261 underwent PCI. Because of this marked group imbalance, the study cannot reliably assess whether ischemia localization is associated with the decision to perform PCI rather than medical management. The principal inferential analysis was therefore restricted to the PCI subgroup and examined whether ischemia localization was associated with the two mutually exclusive patient-level procedural categories recorded in the source dataset (Stent versus PTCA + stent). Using the complete 13-category table and an exact test appropriate for sparse cells, no statistically significant overall association was identified (Monte Carlo exact *p* = 0.7843).

The clinical reasons why the seven patients were managed medically were not systematically available in the retrospective dataset. Potential explanations such as lack of suitability for PCI or consideration of CABG therefore cannot be confirmed and were not used to explain the observed treatment distribution.

Myocardial perfusion imaging remains a widely used non-invasive technique for evaluating myocardial ischemia and supporting risk stratification and management in patients with suspected or known coronary artery disease [4,5]. Contemporary guidelines emphasize the integration of functional ischemia assessment with clinical and anatomical information when considering invasive evaluation or revascularization [1,2]. Observational MPI studies have linked the extent of inducible ischemia to prognosis and to the relative outcomes of medical versus revascularization strategies [7,8].

Most previous MPI studies have focused on ischemic burden, prognosis, or the decision to proceed to coronary angiography or revascularization, whereas relatively few have examined how regional perfusion information relates to subsequent procedural decision-making [7,8,12]. In our cohort, the analysis was therefore focused on the procedural categories actually recorded in the dataset rather than on the decision between medical and invasive management. The findings should be interpreted as an exploratory evaluation of local procedural selection and documentation patterns and not as evidence that a particular ischemic territory increases the need for PCI.

Anterior and anteroseptal myocardial segments are commonly assigned to the left anterior descending (LAD) coronary territory, but this correspondence is not absolute [9,10]. In particular, anterolateral segments may be supplied by the left circumflex artery (LCx), and territorial assignment may vary with coronary dominance and overlap between vascular territories [9,10]. Left-main and bifurcation anatomy can further influence ischemic distribution and procedural planning [14]. Perfusion abnormalities may also arise from coronary microvascular dysfunction rather than a discrete myocardial-vessel lesion [15], while breast/anterior and diaphragmatic/inferior attenuation may alter the apparent regional distribution when artifact adjudication is not specifically documented [4,5]. Accordingly, MPI localization should not be treated as a one-to-one surrogate for a specific culprit vessel, coronary anatomy, or lesion severity. In the present analysis, anterior involvement was not significantly associated with the recorded PCI procedural category after adjustment for age and sex.

Previous studies have shown that greater ischemic burden and integrated anatomic-functional imaging can influence subsequent angiography or revascularization decisions [7,8,12]. These observations provide relevant clinical context; however, they do not establish that ischemia localization itself determined whether PCI was performed in the present cohort, particularly given the very small medical-therapy group and the absence of lesion-level angiographic data.

In the complete PCI dataset, descriptive differences were observed across individual ischemia categories, but the overall 13 × 2 exact test was not statistically significant. Accordingly, no individual regional pattern should be interpreted as favoring one recorded PCI procedural category over the other on the basis of these data alone.

Contemporary revascularization guidance emphasizes that PCI technique is selected according to coronary anatomy, lesion morphology and complexity, vessel size, physiological lesion assessment when appropriate, device characteristics, and procedural considerations [1,14]. Because detailed coronary anatomy, lesion complexity, vessel diameter, physiological lesion assessment, and operator-level factors were not systematically available in the present retrospective dataset, these potential determinants could not be incorporated into the multivariable model.

The lack of a significant association is clinically plausible because procedural strategy is influenced by factors not captured by MPI localization alone. In addition, the present dataset contains broad patient-level procedural labels rather than detailed lesion-level procedural sequences. The categories Stent and PTCA + stent should therefore be interpreted strictly according to the original source documentation and not as a complete characterization of interventional technique; in particular, PTCA + stent is not equivalent to balloon-only PTCA.

Multivariable logistic regression restricted to the PCI subgroup did not demonstrate a statistically significant independent association between ischemia localization and recorded procedural category. Anterior involvement (adjusted OR 1.02, 95% CI 0.58–1.82; *p* = 0.935) and multiregional ischemia (adjusted OR 0.69, 95% CI 0.33–1.41; *p* = 0.304) were not significant after adjustment for age and sex. The post hoc sensitivity analysis that additionally classified ALL as anterior involvement did not materially alter these estimates. Male sex was associated with lower adjusted odds of the Stent category relative to PTCA + stent (adjusted OR 0.40, 95% CI 0.23–0.70; *p* = 0.0012); because sex was a secondary covariate rather than the primary study question, this finding should be considered exploratory.

These findings should be interpreted cautiously. The principal result is negative: ischemia localization did not significantly distinguish between the two recorded PCI procedural categories. The significant sex association may reflect unmeasured differences in coronary anatomy, lesion characteristics, procedural practice, or other confounding factors and should not be interpreted causally.

The clinical implications of these findings are limited. Important limitations include the retrospective design, the very small medical-therapy group, the lack of baseline clinical risk-factor data beyond age and sex, and the coarse patient-level procedural coding. The source screening frame could not be reconstructed beyond the 328 records available for analysis, a distinct normal/no-ischemia MPI category was not preserved, and the intermediate pathway from MPI to coronary angiography was not systematically represented. The exact interval between MPI and the subsequently recorded treatment/procedure was also not systematically available; therefore, a uniform MPI-to-PCI time window could not be imposed retrospectively. Detailed angiographic variables requested for lesion-level interpretation—including stenosis severity and distribution, the number and identity of diseased or treated vessels, proximal LAD or left-main involvement, lesion complexity, vessel diameter, physiological lesion assessment such as FFR/iFR, and concordance between the ischemic territory and the treated coronary vessel—were not systematically available and therefore could not be incorporated into the analysis. The source procedural labels did not provide lesion-level definitions sufficient to equate Stent with direct stenting or PTCA + stent with a standardized balloon-plus-stent sequence; they should therefore be interpreted only as recorded patient-level categories. Standardized segment-level 0–4 stress/rest scores and the derived SSS, SRS, and SDS values were unavailable, so ischemic burden could not be quantitatively assessed. Detailed reconstruction metadata, structured reader-level variables, and structured documentation of caffeine/methylxanthine abstinence before dipyridamole stress were not systematically retained. Dedicated information on attenuation correction, prone imaging, gated wall-motion assessment for artifact adjudication, or formal adjudication of breast/anterior and diaphragmatic/inferior attenuation was also not systematically available. Because ischemia localization was the primary imaging exposure, this represents a potentially important source of regional misclassification. Several ischemia categories were sparsely represented and confidence intervals for regional predictors were relatively wide; therefore, the study may have limited precision and ability to detect modest regional associations. No multiplicity adjustment was applied to secondary exploratory covariates, including sex, so such findings should be regarded as hypothesis-generating. Clinical outcomes—including mortality, myocardial infarction, symptom relief, functional improvement, and quality of life—were not evaluated. In addition, the data reflect local practice from 2013 to 2015; stent technology, physiology-guided lesion assessment, guideline-directed medical therapy, and revascularization strategies have evolved since that period, limiting direct generalizability to contemporary PCI practice. Accordingly, the available data do not support using ischemia localization alone to select one PCI procedural category over another, nor do they allow conclusions regarding the need for PCI, treatment efficacy, or clinical outcomes.

## 5. Conclusions

In conclusion, among patients undergoing PCI, this study did not demonstrate a statistically significant association between ischemia localization and the recorded procedural category (Stent versus PTCA + stent) on either the overall exact test or multivariable analysis. Anterior involvement and multiregional ischemia were not statistically significant predictors of procedural category in the exploratory model. A secondary association with sex was observed and requires confirmation in other datasets. Because only seven patients received medical therapy alone, the present study does not permit reliable conclusions regarding the likelihood of PCI versus medical management or the comparative efficacy of treatment approaches.

Further prospective multicenter studies with larger patient populations are needed to clarify the role of ischemia localization in guiding treatment selection.

## Figures and Tables

**Table 1:** Patient Selection and Study Flow.

Study Flow	*n*
**Patients assessed for eligibility**	**328**
**Excluded**	**60**
− Missing or non-evaluable ischemia localization	41
− Incomplete essential patient/demographic records	16
− Missing treatment information	3
**Final study population**	**268**
Medical therapy alone	7 (2.6%)
Percutaneous coronary intervention	261 (97.4%)

**Table 2:** Baseline Characteristics of the Complete Study Cohort and PCI Procedural Subgroups.

Characteristic	Overall (*n* = 268)	Medical (*n* = 7)	Stent (*n* = 144)	PTCA + Stent (*n* = 117)	*p* Value *
Age, median (IQR), years	67.0 (60.0–73.0)	68.0 (65.5–71.5)	67.5 (60.0–73.3)	66.0 (60.0–73.0)	0.707
Male sex, *n* (%)	150 (56.0)	5 (71.4)	66 (45.8)	79 (67.5)	0.00047
Female sex, *n* (%)	118 (44.0)	2 (28.6)	78 (54.2)	38 (32.5)	-

Values are shown for the complete study cohort and by recorded treatment/procedural category. * Because the medical-therapy group was very small (*n* = 7), inferential baseline comparisons were restricted to the two PCI procedural groups. Age was compared using the Mann–Whitney U test and sex distribution using Fisher’s exact test; exact *p* values are reported where applicable. Diabetes mellitus, hypertension, dyslipidemia, smoking status, prior myocardial infarction, left ventricular ejection fraction, and other cardiovascular risk-factor variables were not systematically available in the source dataset and therefore are not presented.

**Table 3:** Complete Distribution of Ischemia Localization and Recorded Procedural Category.

Ischemic Region	Medical	PTCA + Stent	Stent	Total
Anterior	0	14	30	44
Anteroseptal	1	15	16	32
Inferior	2	28	34	64
Inferolateral	1	22	23	46
Anterior + inferior	0	11	9	20
Apex	0	5	3	8
Apex + inferior	1	3	2	6
Lateral	1	4	4	9
Anterolateral	0	5	9	14
Anterior + apex	1	5	5	11
Inferoseptal	0	3	5	8
ALL	0	2	2	4
Anterolateral + apex	0	0	2	2
**Total**	**7**	**117**	**144**	**268**

## Data Availability

The raw data supporting the conclusions of this article will be made available by the authors on request.
